# Complexities of Severe Leptospirosis: A Case With Acute Hypoxic Respiratory Failure, Acute Kidney Injury, and Hyponatremia

**DOI:** 10.7759/cureus.66027

**Published:** 2024-08-02

**Authors:** Hamza Maqbool, Waqas Memon

**Affiliations:** 1 Internal Medicine, Continental Medical College, Lahore, PAK; 2 Internal Medicine/Nephrology, University of Virginia, Lynchburg, USA

**Keywords:** continuous venovenous hemofiltration (cvvhf), continuous venovenous hemodialysis (cvvhd), continuous renal replacement therapy, rhabdomyolysis, severe isotonic hyponatremia, thrombocytopenia, hyperbilirubinemia, multiorgan failure, weil's disease, leptospirosis

## Abstract

Leptospirosis, an acute zoonotic infection caused by spirochetes of the genus Leptospira, poses significant health risks worldwide. Transmission occurs through contact with infected animals' urine, blood, or tissue. This case report examines a 44-year-old man with severe leptospirosis, presenting as Weil's disease, characterized by acute hypoxic respiratory failure and acute kidney injury (AKI) secondary to rhabdomyolysis, complicated by severe hyponatremia. The case underscores the diagnostic and management challenges associated with leptospirosis, highlighting the importance of interdisciplinary collaboration and comprehensive diagnostic evaluation.

## Introduction

Leptospirosis, a globally distributed zoonotic disease, is caused by the pathogenic spirochete Leptospira interrogans, primarily transmitted by rodents, particularly rats. The main reservoirs of the bacteria are rodents, with urinary excretion being the primary mode of transmission. While most cases present with mild, non-jaundiced febrile symptoms, a minority progresses to a severe form known as Weil's disease, characterized by multiorgan dysfunction. Weil's disease, also termed severe leptospirosis or Weil's syndrome, encompasses a spectrum of symptoms, including high fever, jaundice, renal failure, hepatic necrosis, pulmonary complications, cardiovascular collapse, neurological changes, and hemorrhagic diathesis. Mortality rates for severe cases range from 10-15%, with renal failure, cardiopulmonary failure, and hemorrhage being common causes of death [1-3]. Pulmonary complications have been increasingly observed, with reported prevalence rates as high as 70% [4]. This study explores pulmonary manifestations in leptospirosis, highlighting recent research on associated lung injury. The challenge in diagnosing and treating leptospirosis lies in its overlapping symptoms with other disorders, often mimicking primary lung diseases. Therefore, this review aims to provide insights for accurate diagnosis, drawing from literature and clinical observations.

## Case presentation

A 44-year-old male presented to the hospital with a 6-day history of headache, muscle and joint aches, and diarrhea, followed by 2 days of nausea, vomiting, and shortness of breath. The symptoms commenced after consuming half of a turkey sandwich and frozen chicken. The patient reported a substantial rat population in his living environment, where he frequently cleaned their excrement. He denied experiencing fevers, chills, rashes, joint swelling, recent travel, or insect bites. Notably, he had a medical history of treated Lyme disease three to four years ago.

**Figure 1:**
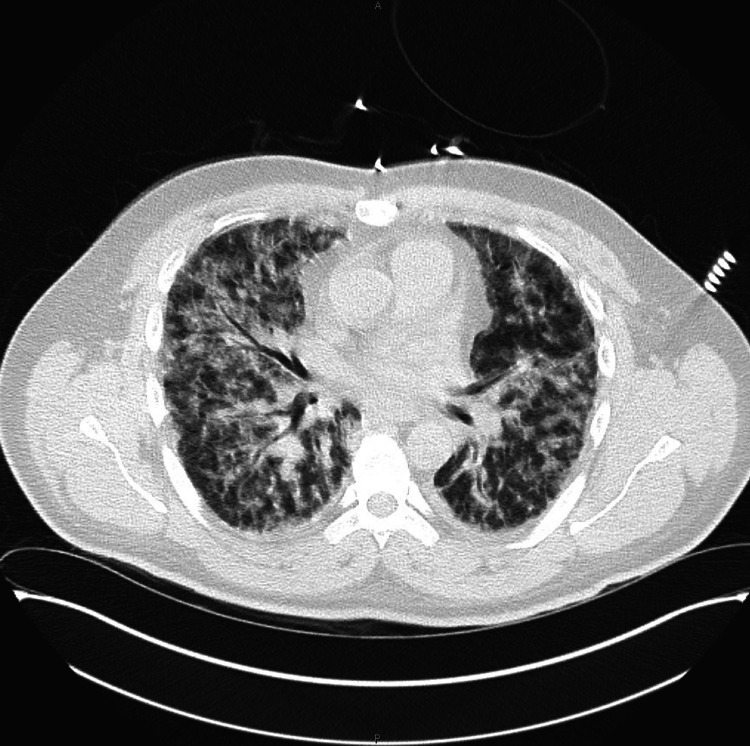
CT chest showing severe progressive diffuse interstitial and airspace lung disease of uncertain etiology The appearance is not typical for community-acquired pneumonia or congestive heart failure/fluid overload.

**Figure 2:**
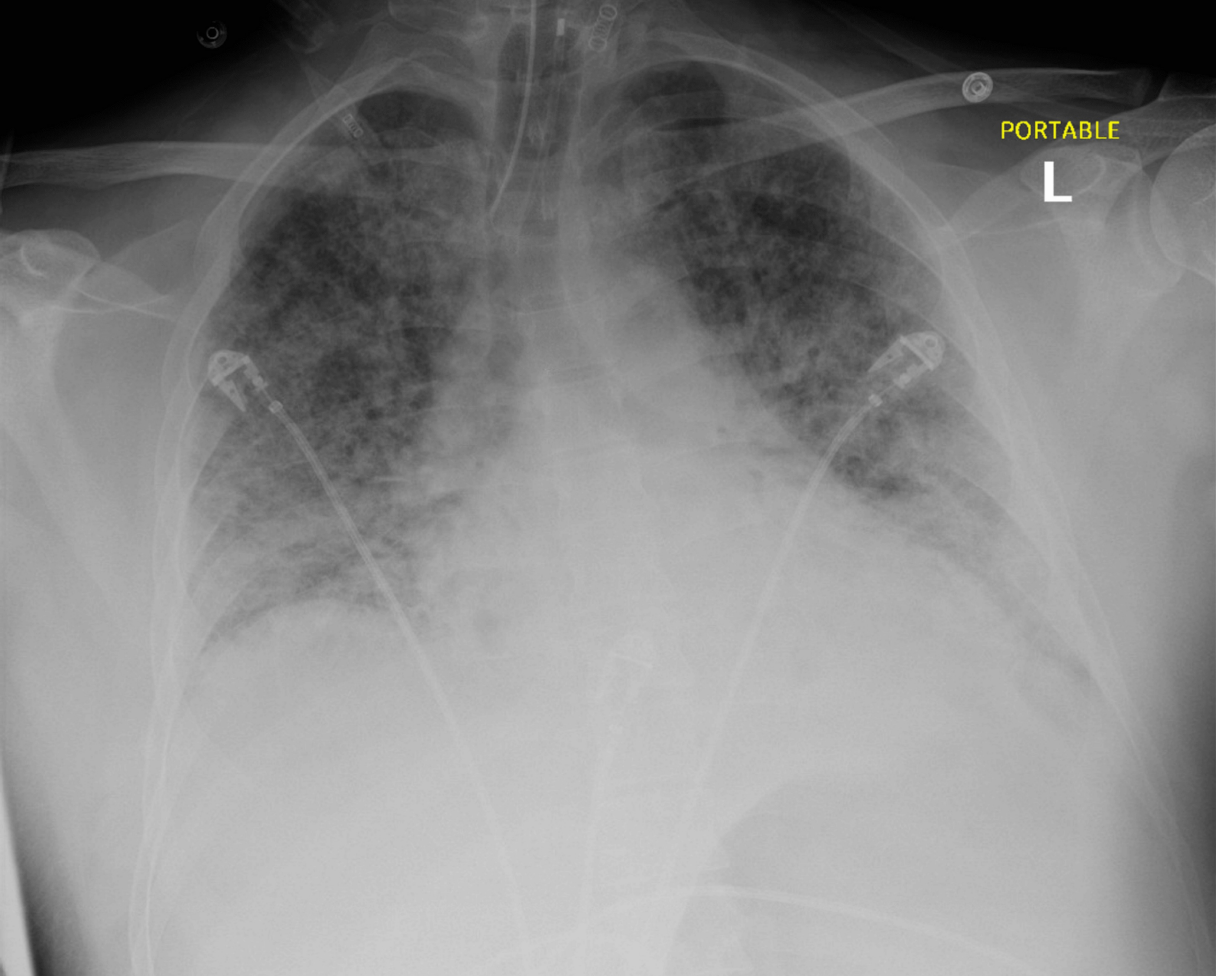
Chest X-ray showing interval intubation with the distal tip of the endotracheal tube projecting 4.4 cm above the carina Diffuse multifocal patchy airspace opacities represent pulmonary edema versus multifocal pneumonia.

On presentation, the patient's vital signs were within normal limits except for a temperature of 99 °F. He was found to be severely hyponatremic (sodium 106 mmol/L) with mild leukocytosis, hemoglobin of 10 g/dL, hematocrit 28.1%, platelets of 45 × 10^9/L, creatinine of 5.5 mg/dL, bilirubin of 4.7 mg/dL, alanine transaminase (ALT) 95 U/L, aspartate aminotransferase (AST) 136 U/L, and creatine kinase (CK) of 23490 U/L. Additionally, he presented with icterus, mild conjunctival hyperemia, nausea, vomiting, decreased appetite, and decreased urine output. Physical examination revealed hyperactive bowel sounds and tenderness to palpation and percussion in the abdomen, without rebound tenderness. Kernig's and Brudzinski's signs were absent. Chest X-ray and CT scan demonstrated bilateral interstitial infiltrates consistent with pulmonary edema and pneumonia (Figures 1, 2).

Differential diagnoses included thrombotic thrombocytopenic purpura-hemolytic uremic syndrome (TTP-HUS), tick-borne illnesses, atypical hemolytic uremic syndrome (HUS), pneumonia, and transaminitis.

Extensive infectious workup was performed, including tick-borne illness serologies, peripheral smear for schistocytes, hemolysis workup, leptospirosis PCR, HIV, hepatitis panel, respiratory viral panel, nasal and throat cultures, and gastrointestinal pathogen PCR. Consultations were sought from Infectious Disease, Hematology, and Nephrology. The patient was initiated on a high-flow nasal cannula for respiratory support and later transferred to the medical ICU due to acute hypoxic respiratory distress. Mechanical intubation, central venous line placement, and bronchoscopy revealed diffuse alveolar hemorrhage. Leptospirosis immunoglobulin M (IgM) PCR confirmed the diagnosis. The patient also developed acute kidney injury (AKI) secondary to rhabdomyolysis and complicated hyponatremia.

Broad-spectrum antibiotics, including doxycycline, azithromycin, and ceftriaxone, were initiated with pathogen coverage for leptospirosis. The patient underwent continuous renal replacement therapy (CRRT). Doxycycline was eventually discontinued, and a nine-day course of ceftriaxone was completed. High-dose steroids were administered (1 gm) to manage the severe presentation. On the fifth day of hospitalization, the patient's renal function, electrolyte imbalances, liver enzymes, anemia, thrombocytopenia, and transaminitis improved, and he was subsequently extubated on the sixth day. The patient gradually improved and was discharged with a tapered dose of steroids, scheduled for follow-up with Infectious Disease within one week.

## Discussion

Leptospirosis, caused by the bacterium Leptospira, exhibits a broad spectrum of clinical symptoms ranging from mild to severe. Among the 21 identified species of Leptospira (L.), L. interrogans and L. biflexa are commonly recognized, with over 200 serovariants [5]. These spiral-shaped organisms, equipped with two flagella, facilitate tissue penetration. Epidemiologically, leptospirosis is a zoonotic disease affecting various hosts, including rats, dogs, wild mammals, fish, and birds. Transmission occurs through direct contact with urine, blood, or infected tissue, as well as indirectly through contaminated water and soil, with human-to-human transmission being rare. The disease is particularly prevalent in tropical regions characterized by frequent floods and poor sanitation, posing a higher risk to specific occupational groups such as veterinarians, farmers, sewer workers, slaughterhouse employees, and those in the fishing industry. Transmission typically occurs through skin abrasions, mucosal exposure, or ingestion of contaminated water. The pathogenesis involves spirochetes entering the body, leading to leptospiraemia, endothelial damage, and subsequent vasculitis. The mechanisms of leptospirosis action are not fully understood, but hypotheses include spirochetal endotoxins binding to toll-like 4 receptors, potentially producing hemolytic toxins and tissue damage. An immune-mediated mechanism via the HLA DQ6 gene and activation of T-lymphocytes is also proposed, along with the involvement of surface lipoproteins like LipL32 in interstitial nephritis [5].

Leptospirosis progresses through two clinical phases: septicemic and immune. Symptoms typically manifest 7 to 12 days post-exposure. The septicemic phase features flu-like symptoms, high fever, headache, myalgias (especially in specific muscle groups), and conjunctival suffusion - a key diagnostic indicator in nonspecific febrile illness [6]. The immune phase follows, with detectable antibodies and leptospires in urine. It involves an immunologic response, lasting over a month. Organ damage may include aseptic meningitis, renal symptoms, and pulmonary manifestations. Elevated liver enzymes and jaundice, pancreatitis, hepatomegaly, and myocarditis can also occur, with liver enzymes indicating prognosis [7].

Severe leptospirosis affects multiple organs, including the kidneys, lungs, liver, and brain. Renal involvement ranges from mild dysfunction to complete failure, such as in Weil’s syndrome. Patients often exhibit acute kidney injury (AKI), possibly due to infection, rhabdomyolysis, and severe isotonic hyponatremia from volume depletion, indicating severe leptospirosis. While most patients with acute renal failure recover, some experience persistent dysfunction with tubular atrophy and interstitial fibrosis. Bleeding is common, with manifestations like petechiae, ecchymoses, and epistaxis, though severe cases may involve gastrointestinal or pulmonary hemorrhage. Thrombocytopenia is frequent but usually not severe enough to cause spontaneous bleeding.

While leptospirosis has been on the rise in developed countries, it remains largely neglected [8]. Lack of awareness among physicians can lead to misdiagnosis or, worse, lack of treatment altogether. Given the highly variable clinical manifestations and severity of leptospirosis, definitive diagnosis relies heavily on serologic tests [9]. Confirmatory tests are often slow, and a delayed diagnosis can have fatal consequences as the disease can progress rapidly [10].

The definitive diagnosis of leptospirosis is established by isolating the organism and observing seroconversion or an increase in antibody titer, typically through the microscopic agglutination test (MAT). A MAT antibody titer ranging from 1:200 to 1:800, consistent with a clinical profile of leptospirosis, provides strong diagnostic evidence. However, significant antibody levels may not be detectable until the second week of infection. The organism can be isolated from serum and/or cerebrospinal fluid within the initial ten days of infection, and from urine for several weeks thereafter [11,12].

The use of antibiotics for severe leptospirosis is debated due to conflicting study results and varying definitions of severity. While some studies show no significant mortality benefit with intravenous penicillin, others suggest a reduced illness duration [13,14]. Current evidence does not strongly support reduced fatality rates when antibiotics are started after the fourth day of symptoms, but most physicians advocate for their use at this stage [15]. Intravenous penicillin is typically administered for 5 to 10 days based on patient response. Empirical antibiotic therapy should begin upon suspicion of leptospirosis [16]. For mild cases, options include oral doxycycline 100 mg twice daily, amoxicillin 500 mg four times daily, or azithromycin 500 mg once daily for three days [8]. In severe cases, treatment may involve intravenous ampicillin (0.5-1 g every 6 hours), penicillin (1.5 million units four times daily), ceftriaxone 1 g once daily, or cefotaxime 1 g four times daily for seven days [8].

**Figure 3:**
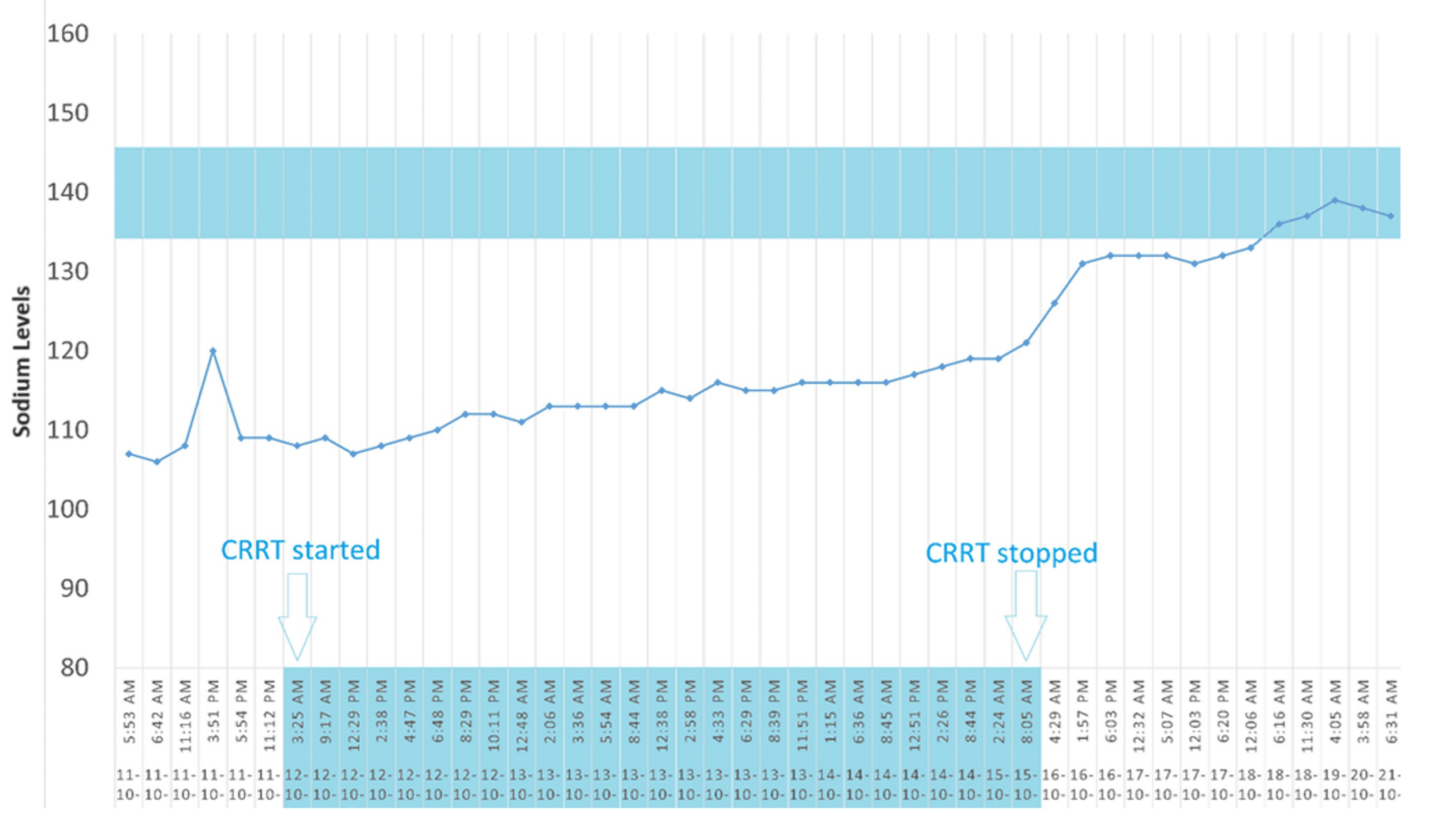
Progression of sodium while on continuous renal replacement therapy (CRRT)

Continuous renal replacement therapy (CRRT) offers the advantage of correcting plasma sodium levels predictably and slowly [17,18]. Unlike standard hemodialysis machines, which are limited to a minimum dialysate sodium concentration of 130 mEq/L, CRRT allows for the customization of sodium levels in the solution, enabling personalized therapy [19,20]. A variety of techniques that differ in their mode of solute clearance may be used, including continuous venovenous hemofiltration with predominantly convective solute clearance, continuous venovenous hemodialysis with predominantly diffusive solute clearance, and continuous venovenous hemodiafiltration, which combines both dialysis and hemofiltration. The initial sodium level for this patient was 106 mEq/L. CRRT was initiated using PrismaSATE 4/2.5, a blood flow rate of 200 ml/hr, ultrafiltration at negative 50 ml/hr, D5W (dextrose 5% in water) replacement fluid at 850 ml/hr, and citrate for anticoagulation. D5W was titrated for sodium goals. Sodium levels were closely monitored, initially fluctuating but gradually improving from 107-120 mEq/L to 111-119 mEq/L, then 121-132 mEq/L, reaching 132 mEq/L, and finally normalizing to 135-145 mEq/L. The gradual correction of sodium levels through CRRT, with careful adjustments and continuous monitoring, successfully managed the severe hyponatremia, highlighting the therapy’s efficacy and the importance of a cautious, tailored approach to avoid rapid osmotic shifts. The total effluent rate is typically reported in milliliters per hour and adjusted by the patient’s weight in kilograms (ml/kg/hr). The determination of the total effluent rate varies according to CRRT modality. In continuous venovenous hemofiltration (CVVH), it is equivalent to the total ultrafiltration rate (the sum of the pre-filter replacement fluid rate, post-filter replacement fluid rate, and the patient’s net fluid removal rate). In continuous venovenous hemodialysis (CVVHD), it is the sum of dialysate fluid rate plus the patient’s net fluid removal rate, and in continuous venovenous hemodiafiltration (CVVHDF) the sum of dialysate fluid rate and total ultrafiltration rate. The equations used for calculating the CVVHD, CVVH, CVVHDF, dilution factor for predilution, total ultrafiltration rate, and plasma flow rate are given in the Appendices. Figure 3 shows the improvement in sodium levels under CRRT.

## Conclusions

This case report highlights the challenges associated with managing severe leptospirosis. The diverse clinical manifestations of this disease can sometimes lead to diagnostic challenges, especially when healthcare providers are less familiar with it or overlook obtaining a comprehensive patient history, including details about the patient's profession or occupation. Early suspicion is crucial in considering leptospirosis as a diagnostic possibility, especially in severe cases, as rapid clinical deterioration can occur, as observed in this patient. Therefore, prompt empirical treatment initiation is essential upon suspicion of leptospirosis to improve patient outcomes.

## Appendices

Equations

1. CVVHD: Dialysate Rate (ml/hr) + Fluid Removal Rate (ml/hr)

2. CVVH: Total Ultrafiltration (UF) Rate (ml/hr) + Fluid Removal Rate (ml/hr)

3. CVVHDF: Total UF Rate (ml/hr) + Dialysate Rate (ml/hr) + Fluid Removal Rate (ml/hr)

4. Dilution Factor for Predilution: Plasma flow rate (ml/hr)/[Plasma Flow Rate (ml/hr) + Pre-Filter Replacement Fluid Rate (ml/hr)

5. Total Ultrafiltration (UF) Rate (ml/hr) = Pre-Filter Replacement Fluid Rate (ml/hr) + Post-Filter Replacement Fluid Rate (ml/hr)

6. Plasma Flow Rate (ml/hr) = Blood Flow Rate (ml/min) x 60 (min/hr) x (1-HCT), where HCT is the current hematocrit of the patient
